# Effect of TiO_2_ Nanoparticles on the Fresh Performance of 3D-Printed Cementitious Materials

**DOI:** 10.3390/ma15113896

**Published:** 2022-05-30

**Authors:** Paulo de Matos, Tuani Zat, Kiara Corazza, Emilia Fensterseifer, Rafael Sakata, Gihad Mohamad, Erich Rodríguez

**Affiliations:** 1 Academic Coordination, Federal University of Santa Maria (UFSM), Cachoeira do Sul 96503-205, Brazil; 2 Department of Structures and Civil Construction, Federal University of Santa Maria (UFSM), Santa Maria 97105-900, Brazil; tuani.zat@acad.ufsm.br (T.Z.); kiara.schneider@acad.ufsm.br (K.C.); emilia.fensterseifer@acad.ufsm.br (E.F.); gihad@ufsm.br (G.M.); erich.rodriguez@ufsm.br (E.R.); 3 Department of Civil Engineering, Federal University of Santa Catarina (UFSC), Florianópolis 88040-900, Brazil; rafael.sakata@posgrad.ufsc.br

**Keywords:** 3D printing, additive manufacturing, cement, TiO_2_ nanoparticles, rheology

## Abstract

3D printing (3DP) of cementitious materials shows several advantages compared to conventional construction methods, but it requires specific fresh-state properties. Nanomaterials have been used in cement-based materials to achieve specific fresh and hardened properties, being potential candidates for 3DP applications. However, there are no reports on using TiO_2_ nanoparticles (nano-TiO_2_) in 3DP cementitious composites. Thus, the current work aims to assess the effect of nano-TiO_2_ on the fresh performance of 3DP cementitious materials. For this purpose, nano-TiO_2_ was incorporated in pastes and mortars from 0 to 1.5 wt.%. Time-resolved hydration (in situ XRD) and rheological and printing-related properties (buildability and printability) were evaluated. Results showed that nano-TiO_2_ particles enhanced the cement hydration kinetics, leading to further ettringite formation up to 140 min compared to plain cement paste. Rheological measurements showed that the nano-TiO_2_ incorporation progressively increased the static and dynamic stress, viscosity, and structuration rate of pastes. Furthermore, nano-TiO_2_ improved the buildability of the composites, progressively increasing the maximum number of successive layers printed before failure from 11 (0 wt.% TiO_2_) to 64 (1.5 wt.% TiO_2_). By contrast, the nano-TiO_2_ addition reduced the printability (i.e., the printable period during which the sample was able to be molded by the 3D-printing process) from 140 min (0% TiO_2_) to 90 min (1.5% TiO_2_). Thus, incorporating “high” nano-TiO_2_ contents (e.g., >1 wt.%) was beneficial for buildability but would require a quicker 3DP process. The adoption of nano-TiO_2_ contents of around 0.75–1.00% may be an interesting choice since it reduced the printability of paste by 30 min compared with the control mix but allowed for printing 24 layers (118% higher than plain mortar).

## 1. Introduction

Three-dimensional printing (3DP) is a digital fabrication process that builds elements by the successive deposition of layers. This technology has been quickly evolving in the last years, reaching scales from a few micrometers for ceramics applications [1] to several meters for building purposes [2]. The use of 3DP in construction brings many advantages, such as increasing productivity, reducing execution errors, building complex shapes, and dismissing the use of formwork, as well as lesser working men [3]. In addition, sustainable binders such as geopolymer [4] and magnesium potassium phosphate cement [5,6] can be used to produce 3D-printed composites with reduced environmental impact.

One of the biggest challenges in 3DP of cementitious materials is to control the rheological properties of the material (i.e., fresh-state behavior). It is known that the rheology of fresh mortar and concrete is governed by the presence, type, and quantity of the constituents (water, binders, aggregates, chemical admixtures, fibers, etc.) [7]. In 3DP applications, fresh material must have relatively low viscosity and moderate yield stress [8]: a low viscosity allows the material to flow through pipes (for printer feeding), but a minimum viscosity is desired to ensure cohesiveness; the yield stress must be enough to provide a stiffness that resists the gravity-induced stress from the above layers [9].

Nanoparticles have been used in cement-based materials mainly to provide new properties (e.g., photocatalytic properties) [10,11,12] or improve the existing ones (e.g., increasing mechanical strength and durability) [13,14]. However, due to the very high specific surface area of nanoparticles, they can also be used to control the rheology of paste, mortar, and concrete. Marchon et al. [15] reported that clay nanoparticles could be used to improve the buildability (addressed in Section 2.3) of 3DP cementitious material by increasing its thixotropy; this improves the shape stability by promoting fresh-state stiffening after printing, while the flowability during printing is not negatively affected. Mendoza Reales et al. [16] also observed that the addition of nanoclay and nanosilica (by up to 1 wt.%) increased the thixotropy of 3DP cement paste. Similar behaviors were reported by Kawashima et al. [17] and Douba et al. [17]. Kruger et al. [18] observed that 1 wt.% of nanosilica increased the static yield stress of mortar by 44%, increasing the maximum printed height before collapsing from 540 to 590 mm. Sanjayan et al. [19] added 0.1–0.3 wt.% nanoclay in mortar, observing progressive increases in the yield stress (by up to 240%), viscosity (by up to 31%), thixotropic index (by up to 156%), and maximum number of printed layers (from 21 to 320) with the increase in the nanoclay content. Senff and co-authors evaluated the effect of nanosilica incorporation on the fresh properties of cement-based materials. The incorporation of up to 2.5 wt.% nanosilica in paste and mortar increased the yield stress (by 66%) and viscosity (by 4%) and decreased the setting time (by 60%) compared to plain cement paste [20]. When nanosilica was coupled with silica fume [21], the authors found that 5 wt.% silica fume addition did not significantly affect the yield stress and viscosity of the mix, while 1 wt.% nanosilica addition increased the viscosity of paste by about 160%; both additions increased the thixotropy of paste, in line with the other reports mentioned above. Nanosilica particles promote a refined pore network as well as longer C-S-H chains and a lower Ca/Si ratio [22]. Yang et al. [23] and Sikora et al. [24] respectively used calcium carbonate and silica nanoparticles to improve the fresh performance of 3DP mixes, discussed in Section 3.

TiO_2_ nanoparticles are often used in cement-based materials for photocatalytic purposes, as mentioned above [10,11,12]. In turn, the fresh-state performance of cementitious materials containing nano-TiO_2_ is much less addressed than materials containing other nanoparticles. Senff et al. [25,26] evaluated the influence of nano-TiO_2_ addition on the rheological behavior of cement paste and mortar. The yield stress and viscosity of pastes increased by 148% and 24%, respectively, when 1 wt.% nano-TiO_2_ was incorporated. Besides, nano-TiO_2_ increased the thixotropy of paste, visually assessed by the hysteresis loop but not quantified. Furthermore, mortar spread (related to yield stress) was reduced by up to 7.4% when 5.2 wt.% nano-TiO_2_ was incorporated. Joshaghani et al. [27] investigated the effect of TiO_2_, Al_2_O_3_, and Fe_2_O_3_ nanoparticles incorporation (0–5 wt.%) on the fresh properties of self-compacting concrete. The authors reported that the TiO_2_-containing concretes required 1.5 times more superplasticizer than plain concrete to reach the required slump flow. Besides, nano-TiO_2_ led to the highest admixture demand despite having the lowest specific surface area among the nanoparticles. Similarly, Jalal et al. [28] assessed the effect of TiO_2_ nanoparticles incorporation (0–5 wt.%) on the fresh performance of self-compacting concrete, observing that the increase in the nano-TiO_2_ content progressively reduced the slump flow (associated with yield stress) by up to 9% and increased the V-funnel time (associated with viscosity) by up to 24% compared to plain concrete.

Although some works investigated the effect of nano-TiO_2_ incorporation on the fresh properties of cementitious materials, this nanomaterial has not been used yet in 3DP mixes, to the best of our knowledge. Thus, the current work investigated the effect of incorporating TiO_2_ nanoparticles on the early hydration, rheology, and printability of cementitious composites. For this purpose, cementitious composites were produced with different nano-TiO_2_ contents, and their fresh performance was evaluated through in situ X-ray diffraction and time-resolved rheological and 3DP tests.

## 2. Materials and Methods

### 2.1. Materials

Commercial Portland cement (Itambé, Belo Horizonte, Brazil), silica fume 971U (Elkem, Oslo, Norway), and ground quartz (Brasilminas, Guarulhos, Brazil) were used for the preparation of the cementitious mortars. TiO_2_ nanopowder A-ZUOJ00133/36 (Tronox, Uerdingen, Germany) in the form of anatase was used, with a median diameter of 223 nm and BET specific surface area of 70.2 m^2^/g. Polycarboxylate-bases superplasticizer (SP) EXP CR 7105 (GCP, Alpharetta, GA, USA) was used, with a solid content of 20.9 wt.% and density of 1.05 g/cm^3^.

**Figure 1:**
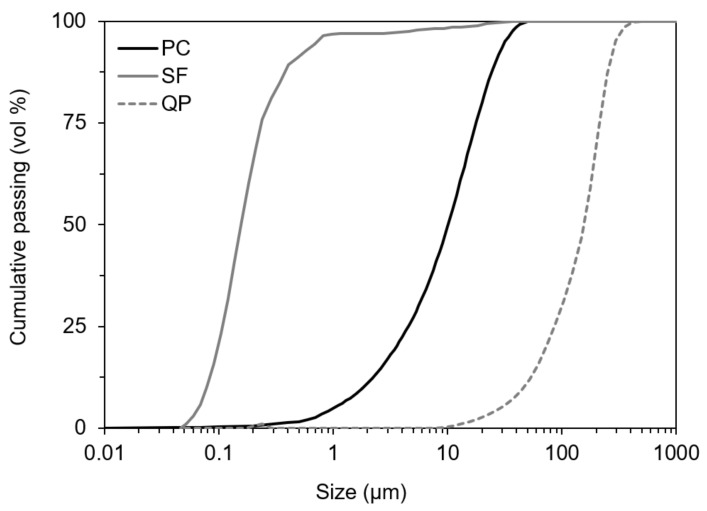
Particle size distribution (PSD) of the Portland cement (PC), silica fume (SF), and quartz powder (QP) used.

Figure 1 shows the particle size distribution (PSD) of the powder materials used determined by laser diffraction, and Table 1 shows the chemical and physical characteristics of the binder fraction determined by X-ray fluorescence (FRX) spectrometry. The mineralogical composition of the cement is presented in Table A1 (see Appendix A), determined by X-ray diffraction (XRD) and Rietveld analysis following Ref. [29].

### 2.2. Mix Proportions and Sample Preparation

A reference mortar was produced without nano-TiO_2_. Its mix proportion was based on 3DP mixes reported in the literature [18,30,31,32,33,34], adopting the following parameters (in weight basis): a water/binder (w/b) ratio of 0.3/1; a PC/SF ratio of 0.9/0.1, and a binder/quartz powder ratio of 1/1. Although the PSD of the quartz powder does not classify it as sand (see Section 2.1), the cementitious composites containing it were called “mortars” to distinguish them from the paste mixes without quartz described next. Then, nano-TiO_2_ was incorporated in cement replacement levels of 0.25, 0.50, 0.75, 1.00, and 1.50 wt.%. These contents were based on previous literature reports that incorporated nano-TiO_2_ in cement-based materials [35,36,37,38,39]. The SP content was adjusted for the reference mix to reach a proper printability and buildability (allowing to print at least 10 layers—see Section 2.3.2), and then was fixed for the other samples. A detailed composition of the mortars produced is presented in Table 2.

In addition, pastes (without quartz powder) were produced for the rheological and hydration tests. The addition of quartz powder in the content present in the mortars would make it difficult to quantify the minor phases of interest (e.g., ettringite) while it exceeded the maximum torque capacity of the rheometer in the rheological tests. The pastes were produced with the same proportions detailed in Table 2 except for the quartz powder.

**Figure 2:**
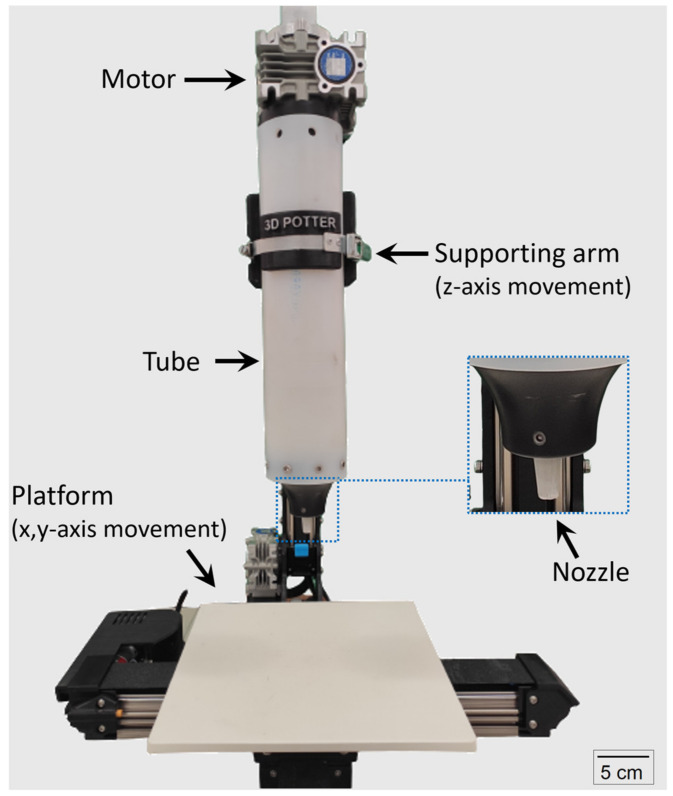
3D printer setup used.

Mortars were prepared in batches of 1 L in a planetary mixer with 5 L and 285 rpm capacity in a controlled room at 23 °C. The mixing procedure was as follows: (i) the dry materials (except for silica fume) were previously homogenized; (ii) the mixer was turned on, and the water + SP (previously mixed) were gradually added over 1 min; (iii) the silica fume was gradually added over 3 min; (iv) the mortar was mixed up to 10 min. Immediately after mixing, the printer container (“tube” in Figure 2) was filled with mortar.

Pastes were prepared in a high-shear capacity mixer in batches of 100 mL. After hand-mixing the dry materials, the powder and the water + SP solution (previously mixed) were added to the mixer container, and the sample was mixed at 10,000 rpm for 2 min.

### 2.3. Testing Methods

#### 2.3.1. 3D Printing—Printability and Buildability

The samples were printed using a Potterbot Micro 10 (3D Potter, Stuart, FL, USA) illustrated in Figure 2, with a nozzle of 6.0 mm in diameter, a layer height of 3.5 mm, and a printing speed of 15 mm/s. This 3D printer is composed of a ram-type extruder where low extrusion rates can promote a slightly bleeding or segregation as a consequence of the high pressure applied [40]. However, more accurate and stiff layers can be printed with this type of extruder when compared to screw-type extruders [41]. The “printability” corresponds to the ability to produce a continuous filament without disruption, while the “buildability” corresponds to the ability to resist successive layer deposition without collapsing. The printability was assessed by printing a continuous filament of 200 mm in length, similar to that proposed by Ma et al. [30]. The buildability was assessed by determining the maximum number of layers printed prior to the overall specimen collapse or the printing of an off-axis (i.e., eccentric) layer when continuously printing a cylindrical specimen of 75 mm in diameter. It is commonly accepted that fresh 3DP elements can fail either by buckling or by the plastic collapse of material [42], leading to a general instability. However, failure can also occur on the scale of a single layer [43], and excessive deformation can cause inadequate layer deposition. This will prevent further layer deposition even though the specimen could support the weight of subsequent layers without abruptly collapsing.

The printability test was repeated every 10 min starting at 30 min of hydration, while the buildability test was performed at only at 30 min of hydration due to material volume limitation. The nozzle was sealed between each printability test to avoid water loss; about 50 mL of sample was discarded before each printing. The buildability test was recorded in video to improve the accuracy of the analysis. The 3DP tests were conducted in a room with a constant temperature of 23 °C.

#### 2.3.2. Fresh-State Tests

Rotational rheometry tests were conducted using a HAAKE MARS III (Thermo Scientific, Waltham, MA, USA) rheometer equipped with a four-blade vane geometry. The vane had 11.00 mm in height and 22.00 mm in diameter, and the cup had 27.20 mm in diameter. The temperature was kept at 23.0 °C using a Peltier unit. After placing the sample in the rheometer container, it was pre-sheared for 60 s at 100 s^−1^ to erase its shear history and rested until starting the rheological measurements.

The rheological properties were measured as follows:

(i) a constant shear rate of 0.01 s^−1^ was kept for 60 s to determine the static yield stress;
(ii) the sample was sheared for 60 s at 100 s^−1^;
(iii) the shear rate was decreased from 100 to 0 s^−1^ during 90 s to determine the flow curve.

The static yield stress (*τ*_0,s_) was defined as the highest shear stress resisted by the material before flowing in (i), while the dynamic yield stress (*τ*_0,d_) and the equivalent plastic viscosity (µ_eq_) were calculated through Equations (1) and (2), respectively, using the data from (iii). The measurements started 30 min after the first contact between the dry materials and water, and the steps (i)–(iii) were repeated every 10 min up to 140 min in the same sample, kept in the rheometer’s container. An isolation hood was used to prevent water evaporation [44].

(1) $$\tau =\tau_{0,d}+K.{\overset{•}{\gamma }}^{n}$$

(2) $$\mu_{\mathrm{eq}\;}=\frac{3K}{n+2}.{\left({\overset{•}{\gamma }}_{max}\right)}^{n-1}$$

where *τ* is the shear stress (Pa), $\overset{•}{\gamma }$ is the shear rate (s^−1^), *K* and *n*, respectively, are the consistency and pseudoplastic parameters of the model, and ${\overset{•}{\gamma }}_{\max}$ is the maximum shear rate applied.

In addition, the structuration rate over time (*A_thix_*) was evaluated using Equation (3) suggested by Roussel and Cussigh [45].

(3) $$\tau_{0,s\left(t\right)}=\tau_{0,s\left(t=0\right)}+t.A_{thix}$$

where *τ*_0,s(*t*)_ is the static yield stress at a given time *t* (Pa), *τ*_0,s(*t*=0)_ is the static yield stress prior to flocculation at a time = 0 (Pa), and *t* is the elapsed time (min).

#### 2.3.3. Cement Hydration

In situ X-ray diffraction (XRD) was conducted using an X’Pert PRO (PANalytical, Almelo, The Netherlands) diffractometer with Bragg–Brentano geometry and an X’Celerator position-sensitive detector. The equipment operated at 45 kV and 40 mA with CuKα_1,2_ radiation. Scans were recorded at the 7–55° 2θ range with a step size of 0.017° 2θ and a total measurement time of 10 min per scan. The measurements started 30 min after the first contact between the dry materials and water and were conducted up to 140 min of hydration. Fresh pastes were placed in the sample holder and were covered with a Kapton foil to avoid carbonation and water evaporation.

Rietveld quantitative phase analysis (QPA) was conducted using TOPAS v5 (Bruker, Billerica, MA, USA) software and the ICSD database (structures detailed in Table A1). The absolute weight fraction of the phases was calculated using the external standard method and the G-factor approach, detailed elsewhere [46,47]. Corundum (α-Al_2_O_3_) was used as external standard also covered with the foil and measured under the same conditions as the fresh pastes. The amorphous contribution of SF was modeled following Naber et al. [48], using an *hkl* phase created through a Pawley fit from a pure SF sample with the tetragonal *P4* space group and fixed lattice parameters *a* = 1.000 Å; *c* = 60.000 Å. The diffuse contributions of free water and the film were also modeled with *hkl* phases as detailed in Refs. [49,50].

## 3. Results and Discussion

### 3.1. Cement Hydration

**Figure 3:**
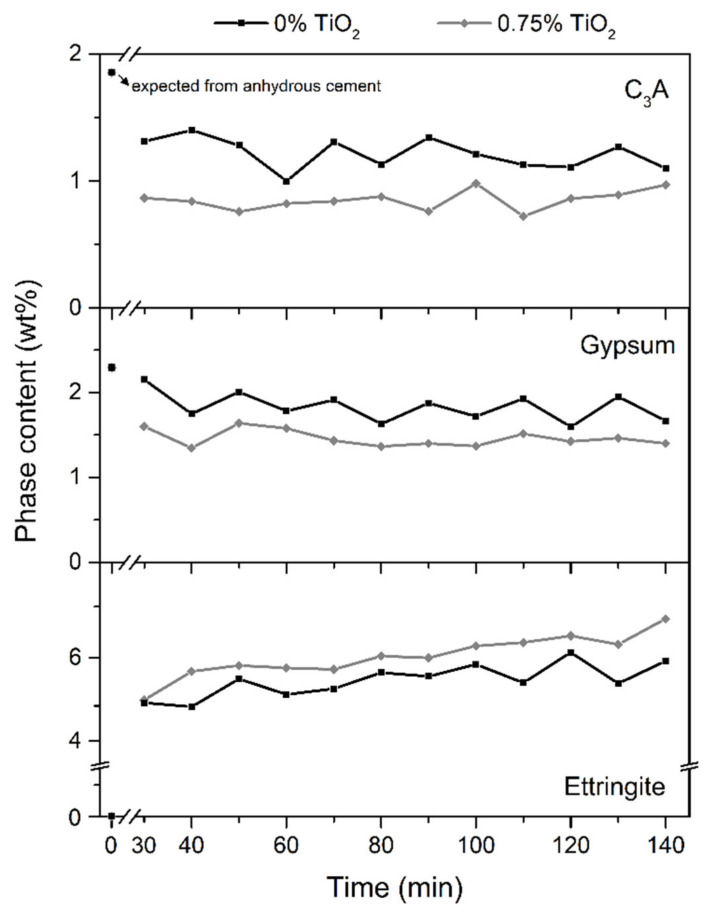
C_3_A, gypsum, and ettringite content of pastes over time obtained by in situ XRD-Rietveld QPA.

Figure 3 shows the C_3_A, gypsum, and ettringite contents over time obtained by in situ XRD and Rietveld QPA for the pastes without nanomaterial (0 wt.% TiO_2_) and with the intermediate content used in this work (0.75 wt.% TiO_2_). The remaining samples were not measured due to equipment access restrictions. The other major phases from the anhydrous cement (C_3_S, C_2_S, and C_4_AF) marginally reacted within the first 140 min of hydration in line with [46,51], so they are not shown. The incorporation of nano-TiO_2_ increased the C_3_A and gypsum consumption and the ettringite precipitation within the first 140 min of hydration. For example, the ettringite content of the 0.75% TiO_2_ mix was about 1 wt% higher than that of the system with 0% TiO_2_ at 140 min of hydration. This trend can be attributed to the very high SSA of the nanoparticles (70.2 m^2^/g), which provide extra surface for the nucleation and growth of hydrated products—here, more specifically ettringite [52]. Hargis et al. [53] demonstrated through in situ soft X-ray images that ettringite can also preferentially grow in nucleation sites such as those commonly observed for C-S-H [52]. Although cement was partially removed to incorporate the nano-TiO_2_, the removal levels were low (0.25–1.50 wt.% in general; 0.75 wt.% for in situ XRD), so factors such as the dilution effect (i.e., the higher water availability due to the increase in the effective water/cement ratio [54]) and the reduced clinker content can be neglected. In turn, it is known that the cement hydration rate is directly related to the surface area of the particles [55]. For instance, the incorporation of 0.75 wt.% nano-TiO_2_ increased the SSA of the binder fraction (i.e., cement + silica fume + nano-TiO_2_) by 12% compared to the reference mix (i.e., from 4.27 to 4.78 m^2^/g), explaining the further reaction degree of the mix containing the nanomaterial at the first 140 min of hydration. In line with our results, Bhojaraju et al. [56] and Wang et al. [57] also reported increased ettringite precipitation within the first hours of hydration in the presence of nanoparticles of graphene oxide and C-S-H, respectively.

Specifically regarding 3DP of Portland-based systems, the early-age hydration enhancement with nanoparticles incorporation was also previously reported. Zhang et al. [33] observed increased heat releases within the first two hours of hydration when incorporated silica fume and/or nanoclay in pastes used for 3DP application. Yang et al. [23] replaced 0–3 wt.% limestone filler with nano-CaCO_3_ in 3DP cement mortars, observing reductions in the induction period and increases in the cumulative heat release in calorimetry (e.g., by 14% at 24 h for 2% replacement) with the incorporation of the nanoparticles. The authors attributed this trend to the extra nucleation sites provided by the high surface area of the nanomaterial discussed above (as expected). This trend can affect the fresh properties of the mixes, as discussed next.

### 3.2. Rheological Characterization

**Figure 4:**
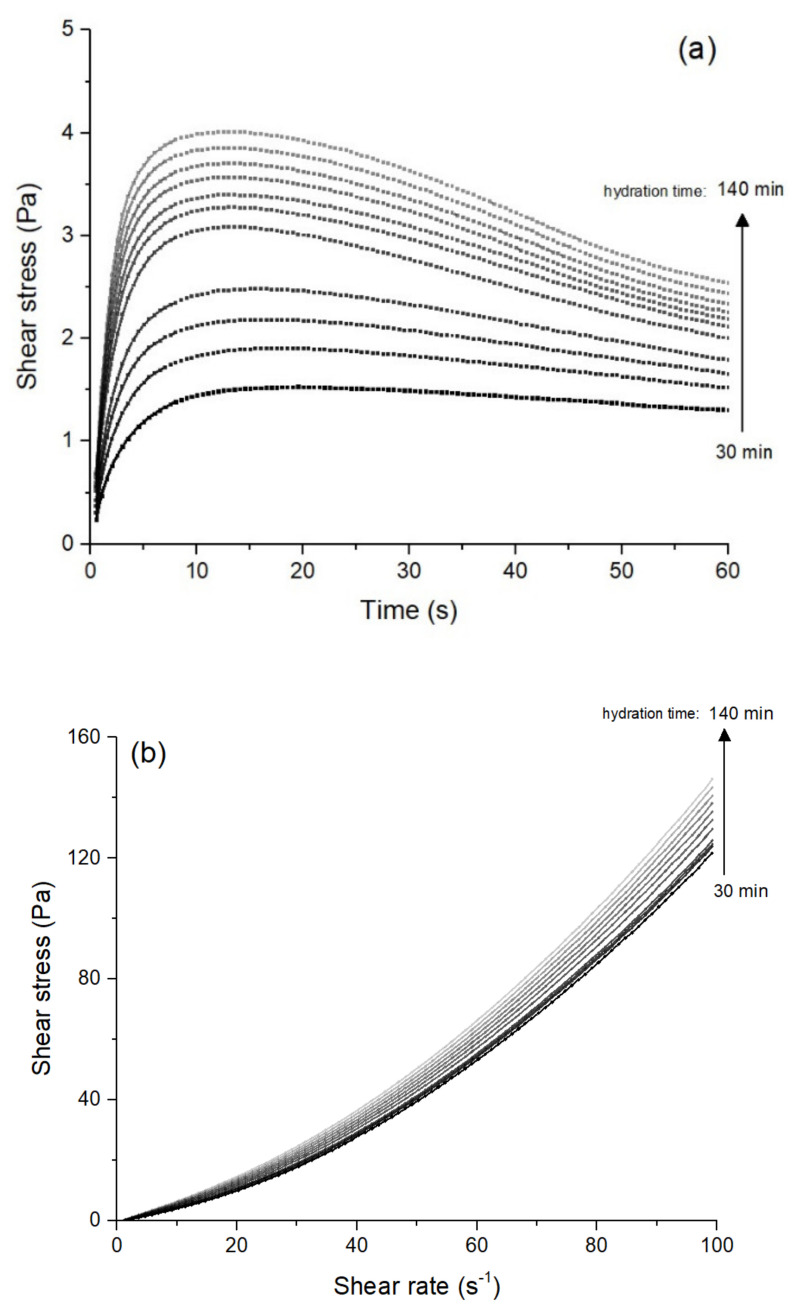
Example of curves obtained in the time-resolved rheological tests for the 0% TiO_2_ mix. (**a**) Shear stress vs. time [constant $\overset{•}{\gamma }$ = 0.01 s^−1^]; (**b**) flow curves.

**Figure 5:**
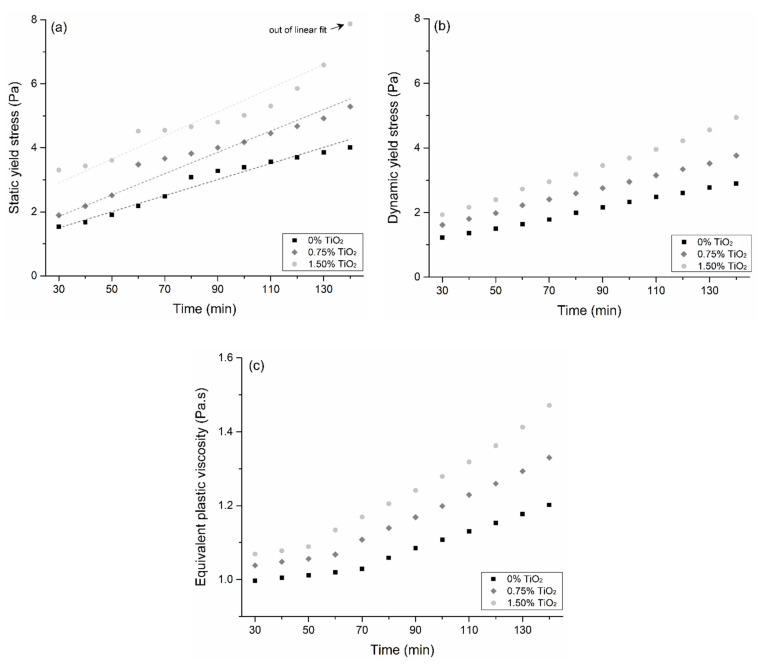
Rheological properties of the pastes over time. (**a**) static yield stress; (**b**) dynamic yield stress; (**c**) equivalent plastic viscosity.

Figure 4 exemplifies the curves obtained in the time-resolved rheological tests for the 0% TiO_2_ mix: the shear stress vs. time in Figure 4a and the flow curves in Figure 4b. Figure 5 shows the rheological properties of the pastes with 0, 0.75, and 1.50 wt.% TiO_2_ over time, and Table 3 shows their structuration rate parameters (*A_thix_*). After 130 min, the static yield stress of the 1.50% TiO_2_ mix grew exponentially, so it was not considered in the linear fit to determine *A_thix_*. It is worth mentioning that the shear stress values found were significantly lower than those reported for 3DP mortars [58,59] due to the absence of aggregates in the samples.

The incorporation of nano-TiO_2_ progressively increased the static and dynamic yield stress, and the viscosity of the pastes for a given elapsed time. For instance, at the first measurement (at 30 min), these increases were up to 116, 85, and 7%, respectively, for the static yield stress, dynamic yield stress, and equivalent plastic viscosity when comparing 1.50% TiO_2_ with 0% TiO_2_. Furthermore, the structuration rate (*A_thix_*) was higher as the nano-TiO_2_ content increased, from 0.025 Pa/min for 0% TiO_2_ to 0.037 Pa/min for 1.5% TiO_2_. The yield stress and viscosity increase with nano-TiO_2_ incorporation, both at the first measurement and over time, can be explained by (*i*) an increased cement hydration rate and (*ii*) a reduction in the PSD of the solid fraction in the nanomaterial presence, discussed next.

Regarding (i), Roussel et al. [60] demonstrated that the time-dependent rigidification of cementitious suspensions occurs due to the combination of flocculation (confirmed by Yang et al. [23]) and the progressive formation of hydration products—more specifically C-S-H, according to the authors—that form a rigid network. In addition, Qian [61] reported that the rigidification rate of a cementitious paste increases with a reduced inter-particle distance due to the higher pseudo-contact between particles and consequently higher C-S-H bridging. In this context, the very high SSA of nano-TiO_2_ reduced the inter-particle distance, consequently increasing the rigidification rate of the cementitious mixes. Although the nanocrystalline structure of C-S-H can be indirectly quantified through XRD (for instance, as suggested by Bergold et al. [62]), this phase is still in the form of colloidal agglomerates at the first hours of hydration [63,64], especially in the presence of SP admixtures [65]. Thus, C-S-H could not be quantified here within the first 140 min of hydration, as observed by [66,67].

Besides C-S-H, ettringite also plays an important role in the rigidification of cement paste since its precipitation consumes a great amount of water (chemical formula Ca_6_Al_2_(SO_4_)_3_(OH)_12_(H_2_O)_26_), and its needle-like shape can hinder the flow [68]. Furthermore, according to Huang et al. [69], the density of ettringite is 1.78 g/cm^3^, while the densities of gypsum and C_3_A are 2.33 and 3.03 g/cm^3^, respectively. Therefore, the increase in the solid volume due to ettringite precipitation is higher than the reduction in the solid volume due to gypsum and C_3_A consumption, naturally increasing the volumetric solid fraction of the system. In this regard, Jakob et al. [70] demonstrated that the ettringite content of a cement paste directly affects its yield stress and viscosity due to the increase in the volumetric solid fraction as a consequence of water consumption by ettringite formation. In agreement with that, our in situ XRD results revealed higher ettringite contents in the mix containing 0.75 wt.% nano-TiO_2_ compared to the reference system (see Section 3.1) during the first 140 min of hydration, which contributed to higher yield stress and viscosity values for the mixes containing the nanomaterial. On the other hand, the presence of nano-TiO_2_ particles can also improve the mechanical performance at early ages, as described by Chen et al. [71] and Meng et al. [38].

As for (ii), Guo et al. [72] demonstrated that the yield stress of a cementitious suspension is closely related to the PSD of its solid fraction: a reduced PSD decreases the inter-particle distance, increasing the probability of collision between particles during flow. In addition, a reduced PSD—and increased SSA—for a given particle concentration increases the amount of water adsorbed into the particles, leaving less water available to lubricate the system. This phenomenon was investigated in detail by Kwan and co-authors [73,74] in terms of water film thickness.

Similar trends of increasing the yield stress and viscosity of cement paste with other nanomaterials incorporation besides TiO_2_ were previously reported by the authors [75,76] and by other research groups [77,78,79]. Specifically for 3DP mixes, Sikora et al. [24] observed that 2% nano-SiO_2_ incorporation was enough to increase the yield stress of 3DP mortars by 4.2 times (from 46 to 239 Pa). Kruger et al. [18] observed progressive increases (up to 76%) in the yield stress of 3DP mortar with 0–3% nano-SiO_2_ incorporation. Yang et al. [23] also reported higher yield stress increases over time for nanoparticle-containing 3DP mixes: the yield stress increased from 30 to 110 min by 39, 322, and 751%, respectively, for 0, 1, and 2% replacement of limestone with nano-CaCO_3_. In turn, specifically regarding nano-TiO_2_ incorporation in cementitious systems, the direct comparison of our results is difficult since most of the existing works only evaluated empirical properties such as slump flow, V-funnel time, and superplasticizer demand [27,28]. Nonetheless, to the best of our knowledge, the only reports that actually measured the rheological properties of the systems are those from Senff et al. [25,26], which also reported increases in the yield stress, viscosity, and thixotropy with nano-TiO_2_ incorporation.

### 3.3. 3D Printing Tests

**Figure 6:**
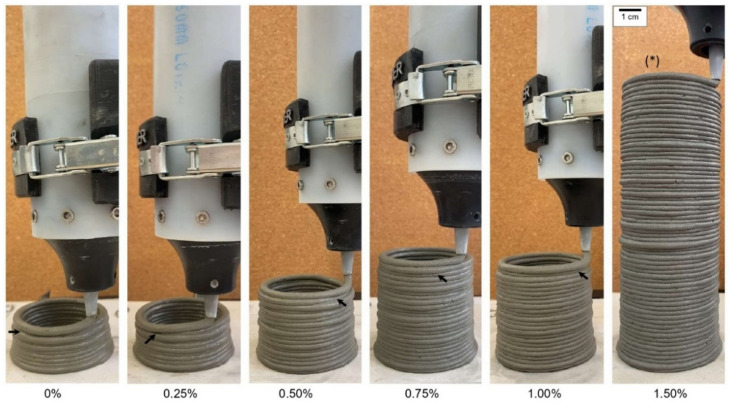
Failure during the buildability test for the different nano-TiO_2_ contents. The arrows indicate the failure; (*) indicates that the failure occurred in the opposite face.

**Figure 7:**
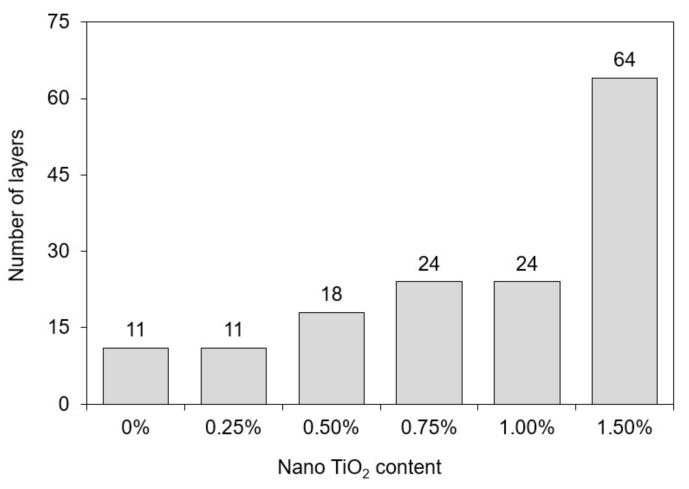
Buildability tests results.

Figure 6 shows the samples during the buildability tests, and Figure 7 shows the buildability results in terms of the maximum number of printed layers before failure. The failure criterion is explained in Section 2.3.1. The incorporation of nano-TiO_2_ progressively improved the buildability of the mixes, increasing the maximum number of printed layers from 11 (0% TiO_2_) to 64 (1.50% TiO_2_). This can be explained by the increase in the yield stress at the first measurement (30 min) promoted by nano-TiO_2_ incorporation, identified by the rheological test results (see Section 3.2). It is well known that freshly printed material must have yield stress that resists the gravity-induced stress from the above layers [41]. By increasing the yield stress of the mixes through nano-TiO_2_ incorporation, the number of printable layers also increased. This relation was confirmed by Yang et al. [23], which simultaneously observed increases in the yield stress and reductions in the vertical displacement of freshly printed mortars (also at 30 min) with nano-CaCO_3_ incorporation, improving their resistance against collapse. For instance, 1% and 2% nano-CaCO_3_ incorporation in limestone replacement reduced the vertical displacement of printed elements by 11.1% and 44.4%, respectively, compared with that containing only limestone. Finally, the yield stress of pastes at 30 min increased by up to 116% with the nano-TiO_2_ incorporation, while the buildability of mortars increased by 4.8 times. Although these two properties are related, the rheological tests were conducted in paste samples, while the 3DP tests were conducted in mortar samples. Therefore, we could not establish a direct correlation between these two parameters.

**Figure 8:**
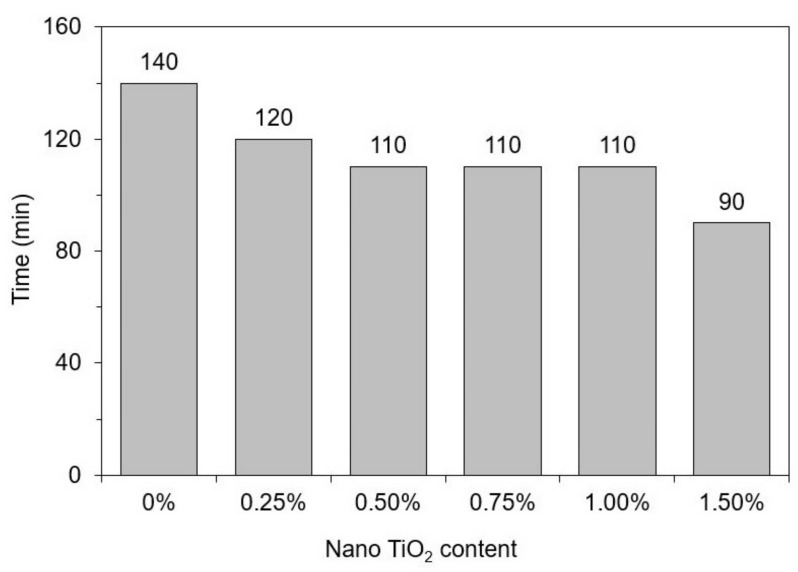
Printability tests results—“open time”.

**Figure 9:**
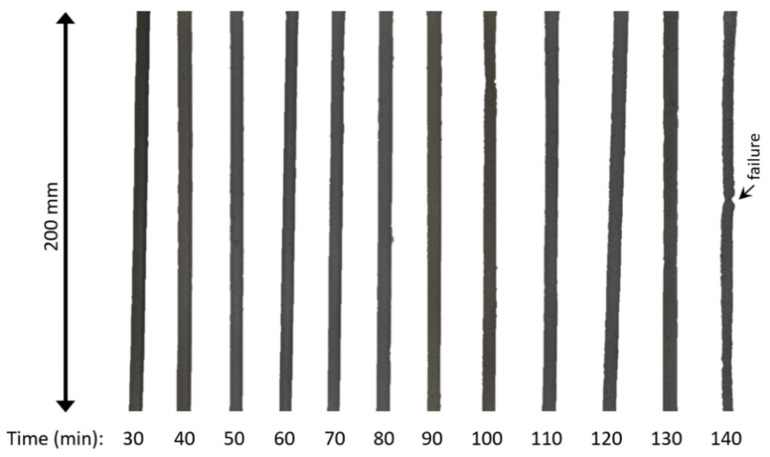
Example of filaments obtained in the printability test (0% TiO_2_).

Figure 8 shows the printability test results in terms of “open time”, defined as the period during which the sample was printable, i.e., it was possible to print a continuous filament with 200 mm in length without any failure, defect, or disruption. Figure 9 exemplifies the filaments obtained in the printability test for the reference mix; all the filaments are shown in Supplementary Materials Figure S1. The incorporation of nano-TiO_2_ progressively reduced the open time of the cementitious composites from 140 min (0% TiO_2_) to 90 min (1.50% TiO_2_). This can be attributed to the higher flocculation and hydration rate in the presence of nano-TiO_2_ (discussed in Section 3.1 and Section 3.2), which increases the rigidification rate of the material and reduces the period that it is “castable” (in this case, printable). Unfortunately, since we could not conduct the rheological test in mortar samples, we were not able to define yield stress and viscosity values that allow suitable 3DP.

One can note that the incorporation of nano-TiO_2_ progressively improved the buildability of the composites (Figure 8) but harmed their printability (Figure 9). This is explained by Souza et al. [2]: on the one hand, the time-dependent stiffening of the material (demonstrated by our rheological test results) makes it harder to print over time; on the other hand, the same phenomenon can improve the mechanical strength of the first layers to support the deposition of the subsequent ones. In this sense, it would be derisible to define a nano-TiO_2_ content that reaches optimum buildability and printability requirements simultaneously. However, this not only depends on the material’s properties but also on the 3DP process (e.g., total printing time, printing speed, size of the printed element, etc.). Nonetheless, as general considerations, we can infer that using “low” nano-TiO_2_ contents (around 0.25%) was not advantageous since it reduced the open time by 20 min without contributing to buildability. Using contents around 0.75–1.00% led to comparable impacts on printability than 0.25% did (with a 10 min difference) but significantly improved the buildability of the mixes, increasing the maximum number of layers by 118%. Finally, the highest nano-TiO_2_ content led to far better buildability, but it would require a quick printing process since it reduced the open time by 50 min compared to the control mix.

## 4. Conclusions

This work investigated the effect of the incorporation of nano-TiO_2_ particles on the fresh-state performance of cementitious composites destinated for 3DP applications. In situ XRD showed that the incorporation of nano-TiO_2_ increased the C_3_A and gypsum consumption and the ettringite precipitation during the first 140 min of hydration. Time-resolved rheological tests showed that the yield stress (both static and dynamic), viscosity, and structuration rate (*A_thix_*) of the pastes increased as the content of nanomaterial increased. This was associated with the reduction in the inter-particle distance due to the very high SSA of nano-TiO_2_ and the increased formation of ettringite (revealed by XRD). As for the printing-related properties, nano-TiO_2_ incorporation improved the buildability of the composites, increasing the number of printed layers before failure from 11 successive layers (0% TiO_2_) to 64 layers (1.5% TiO_2_) at 30 min of hydration. In this sense, nano-TiO_2_ is an effective “thickener” material for the production of cementitious composites destinated to 3D printing applications. In turn, the nanomaterial incorporation progressively reduced the open time (i.e., the period during which it was possible to print a continuous filament of 200 mm in length) from 140 min (0% TiO_2_) to 90 min (1.5% TiO_2_). Thus, incorporating “high” nano-TiO_2_ contents (e.g., >1 wt.%) was beneficial for the buildability of the 3DP composites but would require a quicker printing process. The adoption of “intermediate” nano-TiO_2_ contents (around 0.75–1.00%) may be an interesting choice since it reduced the printability of the composites by 30 min but increased their maximum printable layers by 13 (118%) compared with the control mix.

## Figures and Tables

**Table 1:** Chemical composition and physical properties of the binder materials used.

Property	Portland Cement	Silica Fume	Nano-TiO_2_
*Chemical composition (wt.%)*			
SiO_2_	16.9	93.9	<0.1
Al_2_O_3_	3.6	0.4	0.4
Fe_2_O_3_	2.6	0.4	<0.1
CaO	68.4	0.3	<0.1
K_2_O	1.1	0.8	0.2
Na_2_O	0.1	1.8	0.1
MgO	2.4	2.0	0.1
SO_3_	4.4	0.1	<0.1
TiO_2_	0.3	<0.1	98.5
P_2_O_5_	0.2	<0.1	0.4
* L.O.I	3.46	-	0.40
*Physical property*			
Density (g/cm^3^)	3.08	2.22	4.23
BET SSA ** (m^2^/g)	2.6	19.3	70.2

* L.O.I: loss on ignition at 950 °C; ** SSA: specific surface area.

**Table 2:** Detailed composition of the mortars produced (by weight).

Mix/Material	Cement	Nano-TiO_2_	Silica Fume	Quartz Powder	Water	Superplasticizer
0% TiO_2_	0.9000	0.0000	0.10	1.00	0.30	0.018
0.25% TiO_2_	0.8975	0.0025				
0.50% TiO_2_	0.8950	0.0050				
0.75% TiO_2_	0.8925	0.0075				
1.00% TiO_2_	0.8900	0.0100				
1.50% TiO_2_	0.8850	0.0150				

**Table 3:** Structuration rate parameters of the pastes.

Mix	$\tau_{0,s\left(t=0\right)}$ (Pa)	*A_thix_* (Pa/min)	Coefficient of Determination (R^2^)
0% TiO_2_	0.75	0.025	0.94
0.75% TiO_2_	0.86	0.033	0.93
1.50% TiO_2_	1.80	0.037	0.87

## Data Availability

Not applicable.

## Supplementary Materials

The following supporting information can be downloaded at: https://www.mdpi.com/article/10.3390/ma15113896/s1, Figure S1: Filaments obtained in the printability test. (a) 0% TiO_2_; (b) 0.25% TiO_2_; (c) 0.50% TiO_2_; (d) 0.75% TiO_2_; (e) 1.00% TiO_2_; (f) 1.50% TiO_2_.

## Appendix A

**Table A1:** Mineralogical composition of the cement used, obtained by XRD-Rietveld QPA.

Phase	ICSD Code	Content (wt.%)
C_3_S-M3	94742	33.62
C_3_S-M1	*	13.13
C_2_S-α’H	81097	0.22
β-C_2_S	81096	8.34
C_3_A-cubic	1841	2.50
C_3_A-orthorhombic	1880	0.34
C_4_AF	9197	6.45
Aphthitalite	26014	0.45
Langbeinite	40989	0.31
Syngenite	157072	1.79
Periclase	9863	0.84
Portlandite	15471	1.46
Lime	75786	0.77
Gypsum	151692	3.09
Bassanite	69060	0.33
Calcite	73446	4.93
Dolomite	10404	0.56
Quartz	174	0.28
Ettringite	155395	-
ACn **	-	20.60
Rwp (%)	-	5.14

* Not in ICSD; structure from [80]. ** ACn: amorphous and crystalline non-quantified, determined by the external standard method.
