# Supplementary material for: Effect of TiO2 Nanoparticles on the Fresh Performance of 3D-Printed Cementitious Materials
Source: Materials (Basel). 2022 May 30;15(11):3896. doi: 10.3390/ma15113896 (PMC9182311; doi:10.3390/ma15113896)
Supplement: Supplementary file 1 [file materials-15-03896-s001.zip › materials-1724308-supplementary.pdf]

Supplementary Material for:

Effect of TiO_2 nanoparticles on the fresh performance of 3D printed cementitious materials

Paulo de Matos [1,*]; Tuani Zat [1]; Kiara Corazza [1]; Emilia Fensterseifer [1]; Rafael Sakata [2]; Gihad Mohamad [1]; Erich Rodríguez [1].

[1] Academic Coordination, Federal University of Santa Maria (UFSM), Cachoeira do Sul, 96503-205, Brazil
[2] Department of Structures and Civil Construction, Federal University of Santa Maria (UFSM), Santa Maria 97105-900, Brazil; tuani.zat@acad.ufsm.br (T.Z.); kiara.schneider@acad.ufsm.br (K.C.); emilia.fensterseifer@acad.ufsm.br (E.F.); gihad@ufsm.br (G.M.); erich.rodriguez@ufsm.br (E.R.).
[3] Department of Civil Engineering, Federal University of Santa Catarina (UFSC), Florianópolis 88040-900, Brazil; rafael.sakata@posgrad.ufsc.br
* Correspondence: paulo.matos@ufsm.br

This supplementary material contains the following information related to the above-mentioned manuscript:

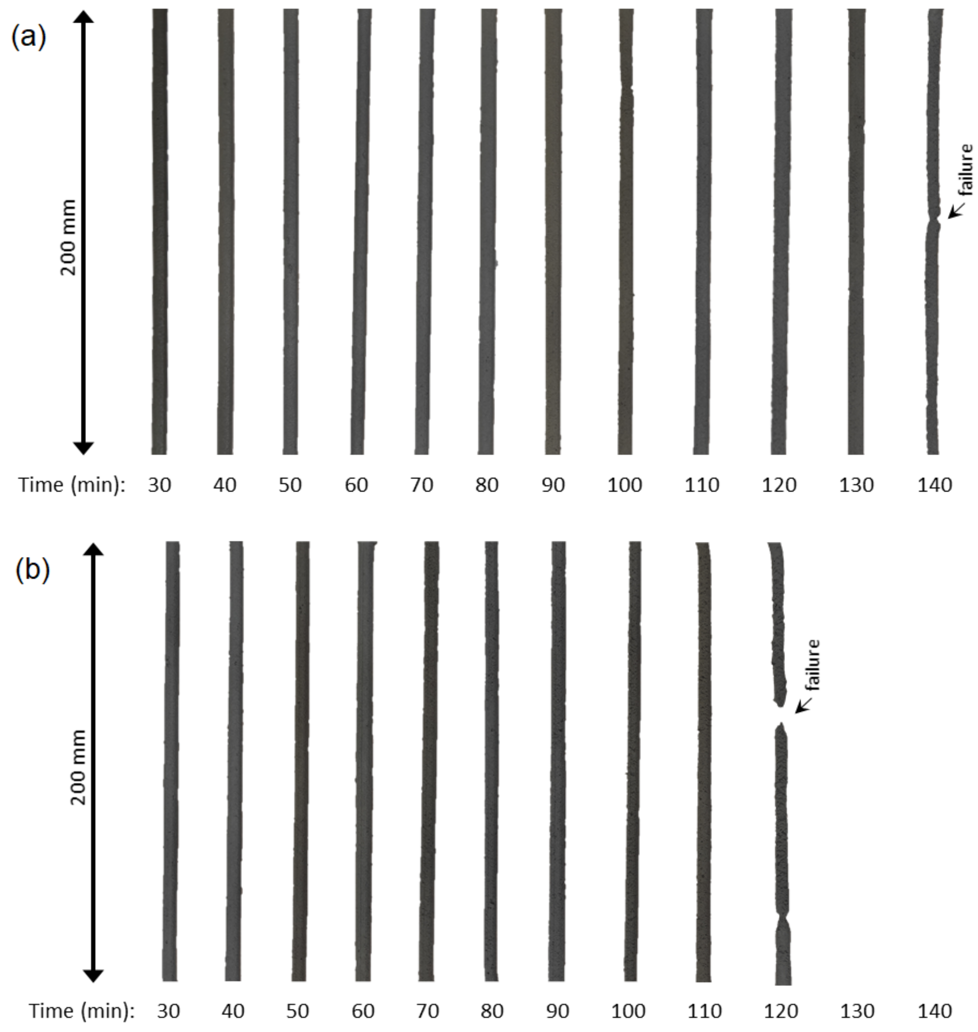

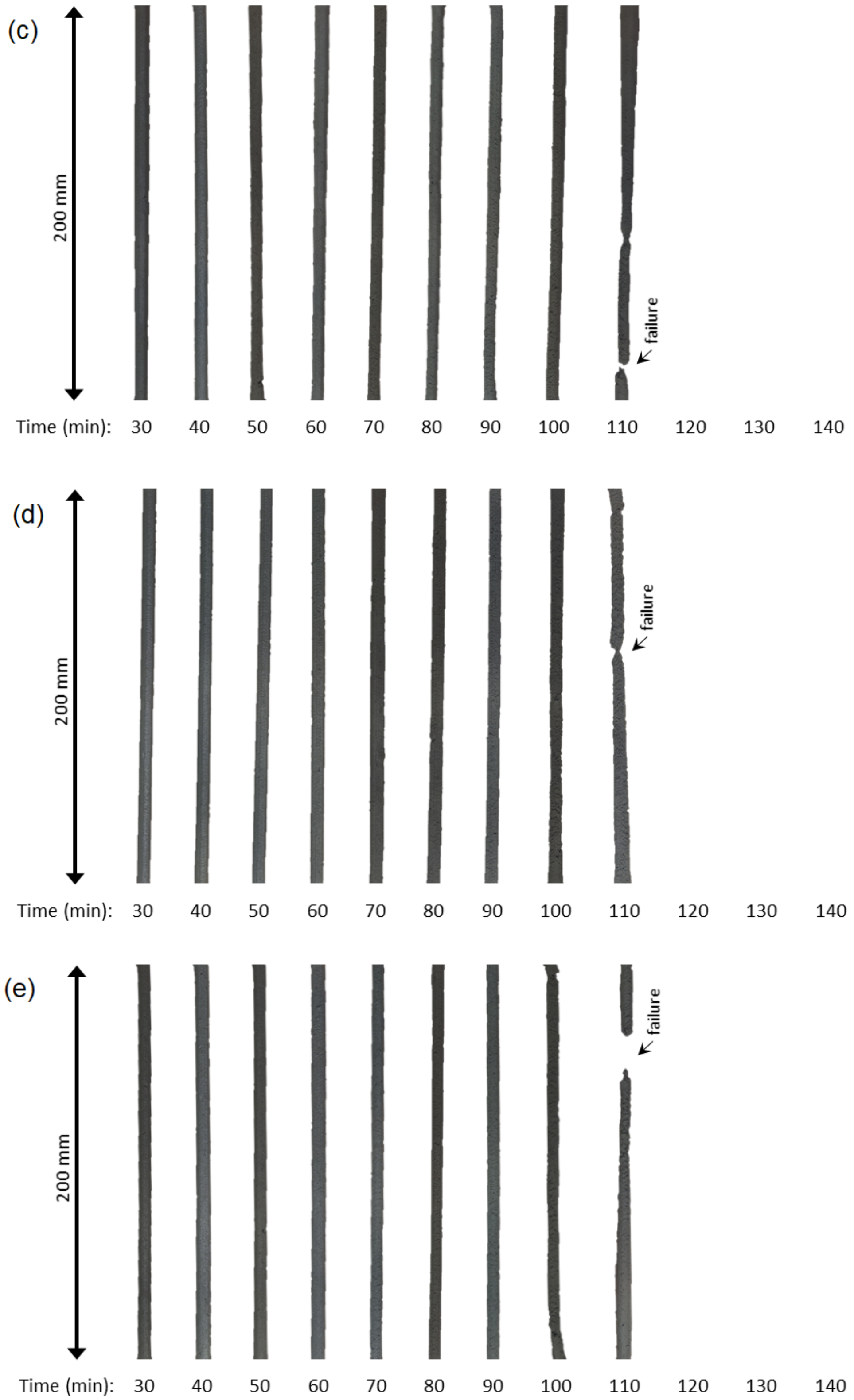

(c)
200 mm
failure
Time (min): 30 40 50 60 70 80 90 100 110 120 130 140
(d)
200 mm
failure
Time (min): 30 40 50 60 70 80 90 100 110 120 130 140
(e)
200 mm
failure
Time (min): 30 40 50 60 70 80 90 100 110 120 130 140

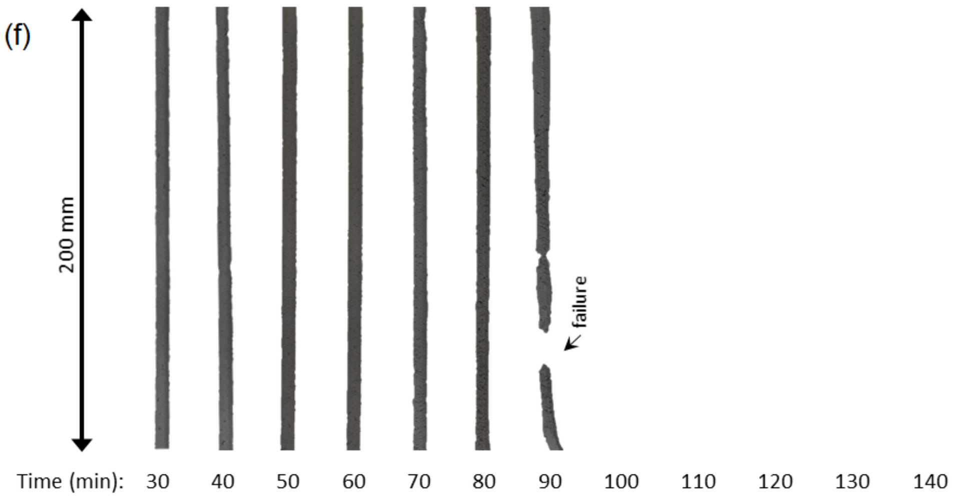

Figure S1. Filaments obtained in the printability test. (**a**) 0% TiO_2; (**b**) 0.25% TiO_2; (**c**) 0.50% TiO_2; (**d**) 0.75% TiO_2; (**e**) 1.00% TiO_2; (**f**) 1.50% TiO_2.
